# Changes in the Floating Plastic Pollution of the Mediterranean Sea in Relation to the Distance to Land

**DOI:** 10.1371/journal.pone.0161581

**Published:** 2016-08-24

**Authors:** Maria Luiza Pedrotti, Stéphanie Petit, Amanda Elineau, Stéphane Bruzaud, Jean-Claude Crebassa, Bruno Dumontet, Elisa Martí, Gabriel Gorsky, Andrés Cózar

**Affiliations:** 1 Sorbonne Universités, UPMC Univ Paris 06, UMR 7093, LOV, Villefranche sur mer, France; 2 CNRS, UMR 7093, LOV, Villefranche sur mer, France; 3 Laboratoire d’Ingénierie des Matériaux de Bretagne, Université de Bretagne-Sud, Rue de Saint Maudé, 56321, Lorient Cedex, France; 4 Expédition MED, 4 Allée des Avettes C.P., 56230, Questembert, France; 5 Departamento de Biología, Facultad de Ciencias del Mar y Ambientales, Universidad de Cádiz, Campus de Excelencia Internacional del Mar, E-11510, Puerto Real, Spain; CSIR-National Institute of Oceanography, INDIA

## Abstract

The composition, size distribution, and abundance of floating plastic debris in surface waters of the Mediterranean Sea were analyzed in relation to distance to land. We combined data from previously published reports with an intensive sampling in inshore waters of the Northwestern Mediterranean. The highest plastic concentrations were found in regions distant from from land as well as in the first kilometer adjacent to the coastline. In this nearshore water strip, plastic concentrations were significantly correlated with the nearness to a coastal human population, with local areas close to large human settlements showing hundreds of thousands of plastic pieces per km^2^. The ratio of plastic to plankton abundance reached particularly high values for the coastal surface waters. Polyethylene, polypropylene and polyamides were the predominant plastic polymers at all distances from coast (86 to 97% of total items), although the diversity of polymers was higher in the 1-km coastal water strip due to a higher frequency of polystyrene or polyacrylic fibers. The plastic size distributions showed a gradual increase in abundance toward small sizes indicating an efficient removal of small plastics from the surface. Nevertheless, the relative abundance of small fragments (< 2 mm) was higher within the 1-km coastal water strip, suggesting a rapid fragmentation down along the shoreline, likely related with the washing ashore on the beaches. This study constitutes a first attempt to determine the impact of plastic debris in areas closest to Mediterranean coast. The presence of a high concentration of plastic including tiny plastic items could have significant environmental, health and economic impacts.

## Introduction

The quantity of plastic entering the ocean from waste generated on land has been recently estimated as on the order of millions of tons per year [1]. Most of the plastic in the waste stream consist of polymers buoyant in seawater such as polyethylene and polypropylene [2], and as such could be potentially transported for long periods by wind and water currents to reach the most remote ocean regions [3–5]. Several surveys have shown the global scale of the marine plastic pollution, with large accumulations of floating debris in distant offshore regions of surface water convergence [6–10]. However, we still know little about the transport and degradation that the floating plastic pollution undergoes from the sources to the accumulation regions. Maritime activities can scatter important amounts of plastic waste over the sea and it seems evident that a fraction of the marine floating debris can be moved onto the coasts by wind and waves [11].

Nevertheless, the nearshore likely make up the main zone of release of plastic, and there is an apparent net transport of floating plastic towards the offshore accumulation zones [4, 5, 8]. Therefore, the distance from coast can be regarded as a proxy of the time at sea of the plastic [12], and the changes in abundance, size or composition of floating plastic debris could help to understand the processes controlling distribution of the plastic pollution in the surface waters.

Small plastic particles can directly enter the ocean as industrial and cosmetic abrasives, pre-production plastic pellets or acrylic textile fibers entering wastewater circuits from clothes washing machines, commonly referred to as primary plastics. Additionally, by the combined mechanical, biological, photic and thermal actions, large plastic objects floating on the sea surface progressively break down into numerous small pieces, referred as secondary microplastic [13]. Surface transport patterns of the marine floating debris are expected to differ in relation to the size or buoyancy of the plastic items [14]. Although marine plastic inputs comprises a heterogeneous assemblage of diverse chemical composition, sizes or colors, this diversity should be modulated over time to select certain characteristics of floating plastic. Plastic debris is removed from the surface by several ways, affecting the plastic sizes and shapes at varying degrees [8, 12]. Critchell et al. [15, 16] modeled the accumulation of marine coastal plastics and suggested that the seeding location and the winds (speeds and directions) are the main factors affecting the accumulation rate of debris on the coastline. In addition, it has been shown that the vertical mixing affects the number, mass, and size distribution of plastics suggesting that that better prediction of winds, vertical transport processes but also ocean plastic properties is needed to correctly quantify distribution and abundance of fragments in the ocean [17,18].

The Mediterranean Sea is a large enclosed basin that supports a strong demographic pressure, with 466 million inhabitants settled on its coasts [19]. The problem of the anthropogenic marine litter was highlighted as an issue of concern in 1976 with the Barcelona Convention for the Protection of the Mediterranean Sea. In 2013, extensive visual surveys across the central part of the basin showed that plastics constituted most of the floating litter, comprising sometimes up to 100% of the debris observed [20]. In this same year, using a basin-scale sampling with surface net tows Cózar et al. [21] demonstrated that the Mediterranean Sea can be regarded as a great accumulation region of plastic with concentrations comparable to those found in the inner accumulation regions of the great Subtropical Gyres. The high load of floating plastic likely results from significant plastic input combined with the specific configuration of the basin and a limited export to the Atlantic Ocean. The patchy distribution suggested that the variability in the Mediterranean surface circulation hampers the formation of stable plastic retention areas. Using multi-annual simulations of the transport of floating debris into the Mediterranean Mansui et al. [22] did not identified permanent structures that may retain floating objects given that the circulation variability led to sufficient anomalies to alter their spatial distribution.

In the present work, we analyzed the floating plastic debris collected by net tows during a survey carried out in 2013 in waters of the Northwestern Mediterranean, especially within the 1-km water strip adjacent to coast, a gap in the previous basin-scale analyses of the Mediterranean [20, 21].

We hypothesized that there are high concentrations of floating plastic debris in the nearshore waters of the Mediterranean Sea. The relatively high concentrations of plastics scattered throughout the Mediterranean [21] together with the leakiness of the offshore accumulations zones due to the circulation unsteadiness and mixing events [22] could lead to plastic accumulation near the coasts in the periphery of the Mediterranean basin.

The aim of this study was to conduct a wider analysis of floating plastic distribution in the Mediterranean through integration of our data with recent net tows data [21, 23], and to characterise relationships of abundance (absolute and relative to plankton), size and polymeric composition in relation to the distance from the land.

## Material and Methods

**Fig 1:**
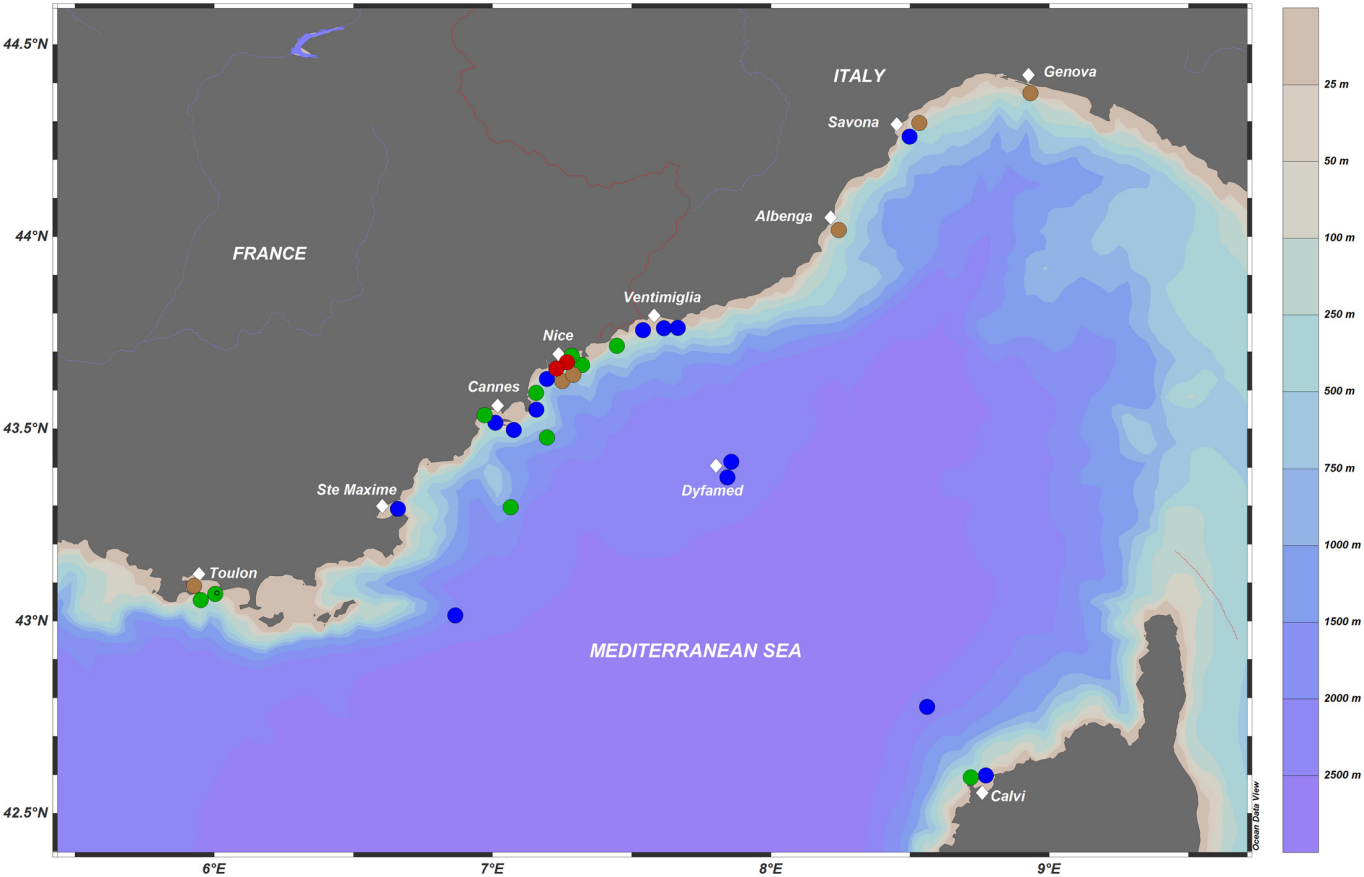
Spatial distribution of the surface plastic concentrations measured in the northwestern Mediterranean in July and August 2013. Blue, green, orange and red, circles represent concentrations of 20–75,000; 75–150,000; 150–500,000 and >500,000 pieces km ^-2^, respectively. Schlitzer, R., Ocean Data View, odv.awi.de, 2015.

Floating plastic debris and surface plankton were sampled in the framework of the participative science activities of Expeditions MED (www.expeditionmed.eu) association from July 6th to August 6th 2013. Sampling was carried out at a total of 33 sites across the Ligurian Sea (NW Mediterranean sea), with distances to land ranging from 0.3 to 46 km. Fifty four percent of the sampling sites were within the 1-km water strip adjacent to the mainland coast. Geographical coordinates and dates of sampling are available at Pangea Data Publisher http://www.pangaea.de and illustrated in Fig 1. Permission for navigation and research operations in exclusive economic zones Sea was granted from the Governments of France and Italy.

Samples were collected using a Manta trawl net with 333 μm mesh size with a rectangular net opening of 60 x 20 cm. The net was towed at the top 10 cm of the sea surface with an average speed of 2.5 knots during c.a. 60 min, corresponding to an average covered area of 3000 m^2^ per tow and an average filtered volume of 371 m^3^. Sampling did not involve endangered or protected species. Net tows were always carried out in calm sea conditions, with Beaufort Sea State varying from 0 to 2, in order to minimize the effect of the wind-induced vertical mixing of the floating plastic debris.

In the lab, samples were gently mixed and transferred into a 2 L glass jar. Floating plastic debris was carefully picked from the supernatant. This process was repeated 3 to 6 times to ensure the removal of all of the smallest floating plastic particles. We also removed plastic particles that aggregated or sedimented with zooplankton and organic matter under a dissecting microscope. Plastic items were counted, and measured by imaging analysis using the Zooscan system [24, 25]. Firstly, they were digitally imaged with a Zooscan digital scanner with a resolution of 2400 dpi; each pixel is about 10 microns wide. Image post-processing was performed with the Zooprocess & Plankton Identifier software that gives a set of morphological parameters for each object, including ferret diameter (i.e. the maximal distance between any two points along the boundary of the object) and surface area (mm). Zooplankton of 30 samples (ranging from 0.5 and 46 km far from land) was also processed, except 3 samples containing too many aggregates to be accurately analyzed with ZooScan. We calculated a plastic plankton ratio by dividing the total concentration of microplastics by zooplankton concentrations for all the 30 stations.

The relationship between plastic concentrations and coastal population densities was performed by consulting the statistical results of the 2012 population census database of the French National Institute of Statistics and Economic Studies (INSEE) listing population categories and their composition [26]. The Italian coastal populations densities, characteristics, and statistics were consulted on the following official website: http://en.comuni-italiani.it.

A total of 407 plastic items were randomly extracted from 28 manta tows to identify their chemical structure by Fourier Transform Infrared Spectroscopy (FT-IR) spectra, using a FT-IR spectrometer (Shimadzu 8400 M) with 4 cm^-1^ resolution and 40 scans. All spectra were recorded in the absorbance mode in the 4000–600 cm^-1^ region. Each sample was put onto the diamond cell and compressed between two plates into a thin uniform thickness enough to allow for adequate transmission of IR beam through the sample to the detector and resulting in a better quality spectrum. FT-IR spectra of the plastic samples were compared with spectra of known plastics to identify polyacrylics (PAA), polyamides (PA), styrenics (homopolystyrene (PS), PS-based copolymers or PS foams) and polymers belonging to the family of the polyolefins, including polyethylene (PE), propylene (PP) and ethylene vinyl acetate (EVA).

To analyze the changes in the plastic pollution in relation to the distance to land, we combined our data of plastic abundances (n = 33 distance to land: 0.3–46 km) with those recently reported by de Lucia et al. for the Central-Western Mediterranean (n = 10; distance to land: 0.5–18 km; [23] and by Cózar et al. for the entire Mediterranean basin (n = 28; distance to land: 7.6–340 km; [21]. Furthermore, to characterize the size distribution pattern of plastics we integrated our data (total of 10,540 plastic items measured) with the data obtained by Cózar et al. (3,812 items measured) in Mediterranean Sea.

Overall, the data compilation of the sampling sites were grouped into three strips, < 1 km from land (8,053 items), between 1 and 10 km from land (1,830 items), and from 10 km to 46 km (4,469 items) in agreement with the local bathymetry and currents. The coastal area of the Ligurian Sea is characterized by a very narrow continental shelf with deep sea near the coast. The zone < 1 km (up 500m depth) corresponded to adjacent costal area under direct influence of fresh anthropogenic litter input. The zone between 1 km and 10 km (~1000 m depth), corresponded to the peripheral zone with mesoscale variability periodically impacted by the geostrophic Northern Current. The area beyond 10 km is under intense mesoscale activity, influenced by the Northern Current and the strong frontal zone separating the peripheral coastal water from central water masses [27, 28].

Plastic items of the three zones were divided into 28 size classes beginning from 0.33 mm (mesh size used in our study) and size limits following a 0.1-log series of linear length, thus using wider bins as plastic items are larger [8]. In addition, we compared the size distributions in the remote areas far from coast (> 10 km), where the number of plastic items was relatively high (n = 3756 in Cózar et al. and n = 712 in this study). The size distributions were in good agreement, with a smoother shape for the sampling with higher data number (S1 Fig). The size distributions were represented as abundance normalized by the number of items collected in each of the three zones. We specifically divided the abundance of plastics in each size class per the total number of large plastics (> 10 mm) to obtain their relative abundances. Using the large-sized plastic items as reference, we analyzed the possible differences in the transfer of plastic from large-size classes toward small-size classes by fragmentation.

## Results and Discussion

Plastic debris was present in all our surface net tows (Fig 1). We found a few pellets/granules and beads, but the predominant plastic forms were basically composed by pieces of films, foam plastic, filaments, and particularly fragments of rigid objects, in agreement with the results found for offshore Mediterranean waters [21]. It seems that the fragmentation of large plastic manufactured objects was the main source of microplastics. The estimated concentrations for each net tow ranged from 2.1 x 10^4^ at the offshore DYFAMED station (43.4167N, 7.8667E http://sodyf.obs-vlfr.fr) located in the central zone of the Ligurian Sea, to a maximum concentration of 5.78 x 10^5^ plastic debris per km^2^ near the coast of Nice (France). The study of individual particles based on ZooScan optical analysis showed a broad size range of plastics ranging from 0.32 to 100 mm with particles displaying an asymmetrical frequency distribution skewed toward smaller size classes. Results showed that across the sampling area, small fragments measuring less than 2.5mm composed 64% of microplastics observed, fragments measuring 2.5–5 mm composed 27% and larger fragments (>5 mm) represented 9% of the total plastic collected. The median length of plastics was 1.95 mm with an increase in the size of fragments with the distance from the coast 1.85, 2.76 and 3.86 mm respectively at < 1 km, 1–10 km, >10–46 km.

**Fig 2:**
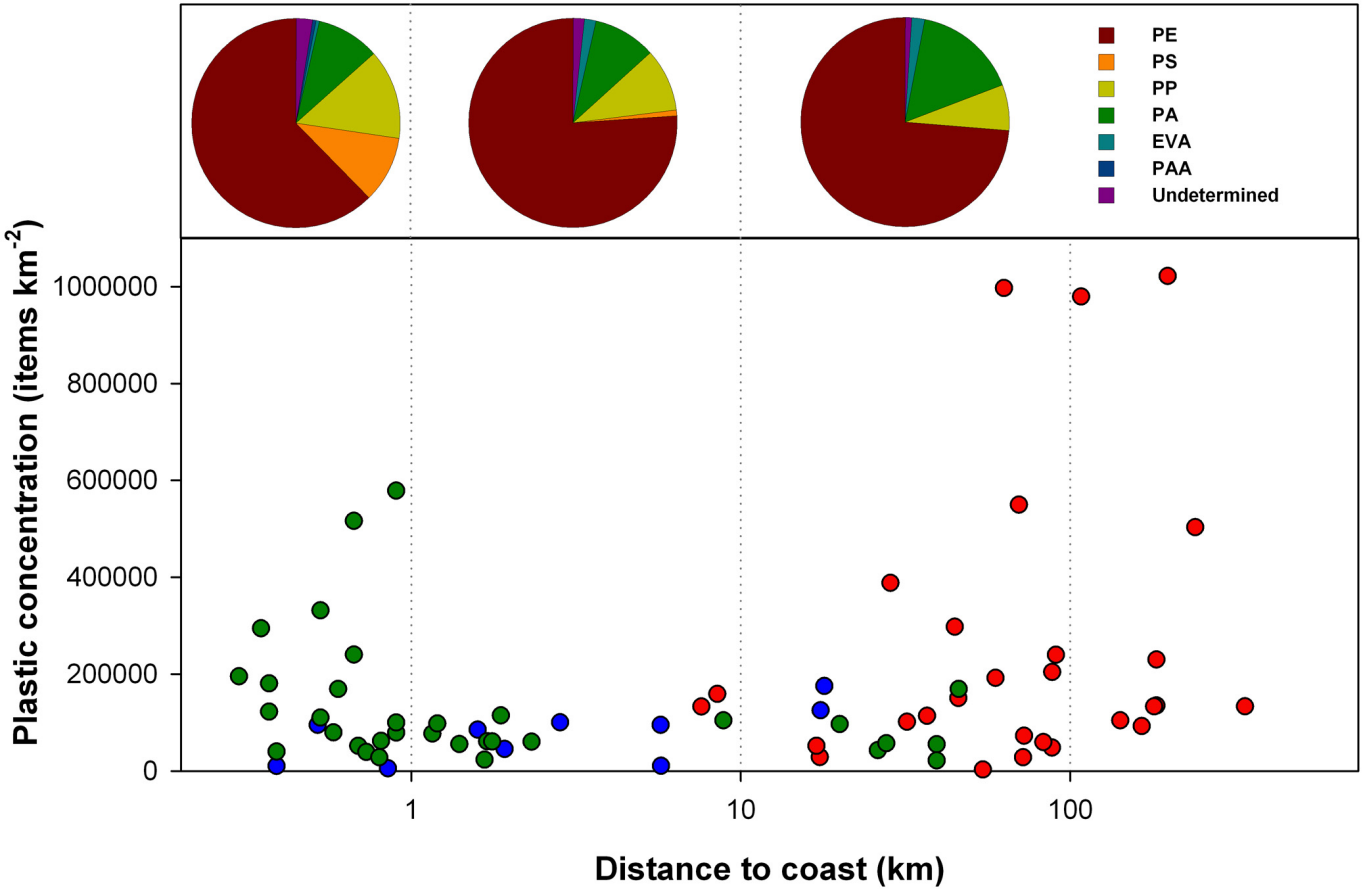
Concentrations and polymeric composition of floating plastic debris in relation to the distance from land in the Mediterranean Sea. Pie charts show the polymeric composition for the three water strips, <1 km (194 items analyzed), 1–10 km (113 items), and >10 km from land (99 items analyzed). Plastic fragments’s abundances (lower graph) includes measurements made during this study in the western Mediterranean (green circles) and data reported by Lucia et al. (blue circles; [17]) and Cózar et al. (red circles; [15]).

**Fig 3:**
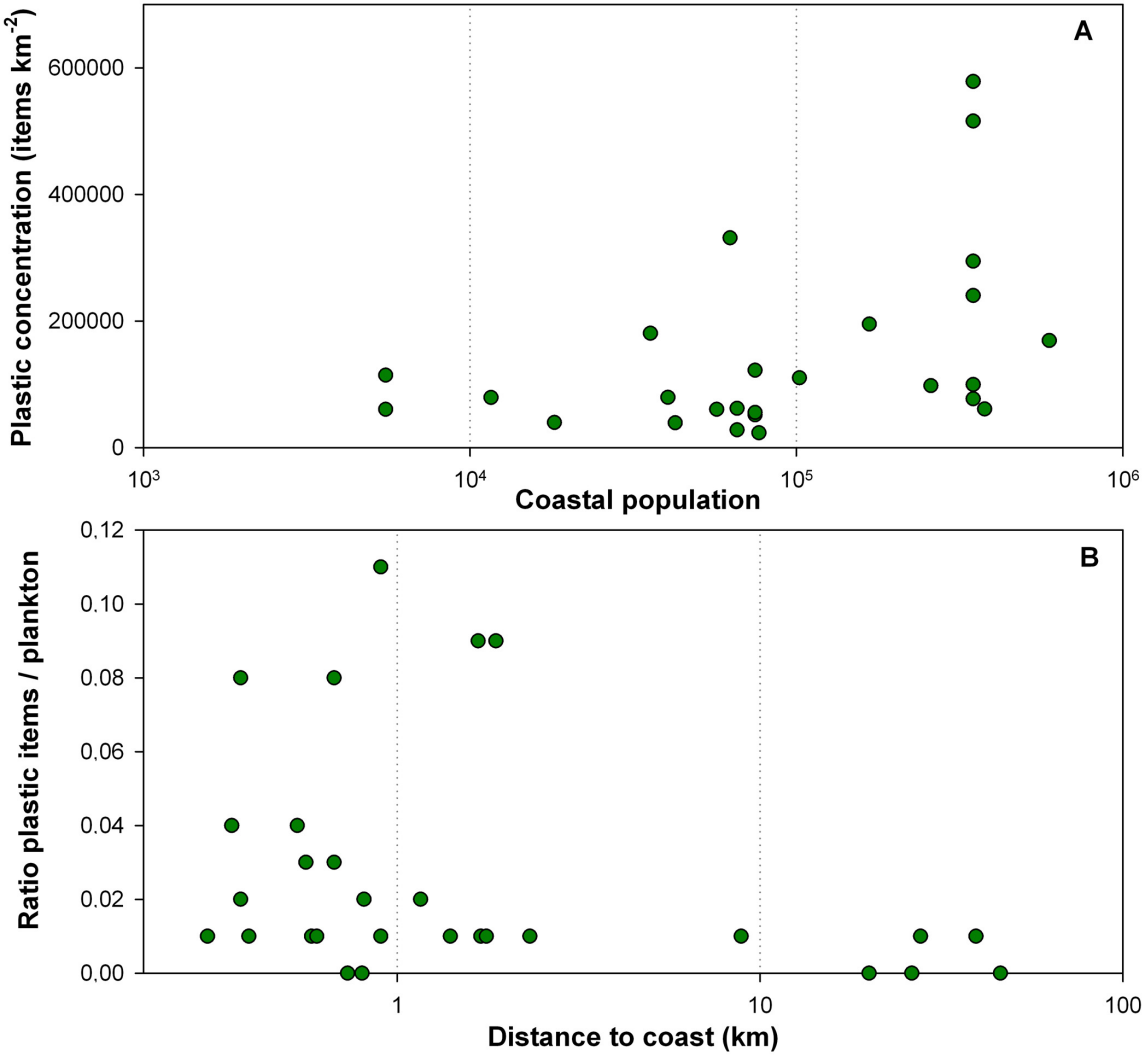
A-Concentrations of floating plastic adjacent to land in relation to the nearest coastal urban center (# of habitants). We considered the urban center presenting more than 5000 inhabitants and the sampling stations situated within the 2-km water strip from these urban centers. B-Plastic to plankton ratios by distance to the coast (km).

The combined data set of our study with those previously reported for the Mediterranean Sea [21, 23], showed higher abundance of plastics towards land and towards offshore regions (Fig 2). High concentrations of plastic debris were commonly measured in the 1-km water strip adjacent to coast, ranging from 28,000 to 578,000 items km^-2^ and with most of the concentrations (56%) above 100,000 items km^-2^. Surface samples generally showed low concentrations in waters between 1 and 10 km from the coast (only 25% above 100,000 items km^-2^), and again reached high values towards distant waters (54% and 89% above 100,000 items km^-2^ for 10–100 km and > 100 km). Aggregating samples by distance to land, < 1 km, 1–10 km, 10–100 km, and > 100 km, the mean and deviation of the plastic concentrations (in thousands of items per sq. km) were 158 ±157, 80 ±38, 176 ±216, and 370 ±378, respectively. The increase in surface plastic concentrations nearshore is expected by the proximity of land-based sources, and the accumulation of floating plastic in the basin periphery follows energetic mixing forces. The spatial heterogeneity in these regions was significantly higher probably due to the combined effect induced by wind and water currents. Previous studies on the spatial distribution of plastic in Mediterranean have referred to these effects as the major cause of the apparent lack of stable plastic retention areas [21, 22]. The uneven distribution of coastal populations and land-based pollution sources could also contribute to the heterogeneity observed in the nearshore waters. However, a significant correlation (R = 0.449, P = 0.010, n = 26) between surface plastic concentrations and the coastal human population was found for the adjacent coastal area (Fig 3A). This suggests that a major part of the coastal plastic pollution may come from the nearby land-based sources and coastal maritime activities linked to densely populated zones, as proposed for other regions around the world [29, 30]. However, in our dataset the differences in numerical concentrations of plastic along the shoreline were due to changes in the abundance of tiny plastic pieces instead of newly introduced large-sized objects. This was supported by the considerably higher relative abundance of small plastic particles (< 2 mm) in the 1-km water strip adjacent to coast. Hence, the residence time of the plastic pollution entering from land within the nearshore waters seems long enough to undergo an important fragmentation. This is agreement with the model of Isobe et al. [14]; they noted that the largest plastics (> 5 mm), being more buoyant, are selectively trapped close to the coast by effect of the Stokes drift produced by the wave. Thus, large plastic objects would be commonly washed ashore on beaches to degrade into small fragments before leaving the nearshore waters.

A data bank containing the spectra of main plastics has been previously established according to the statistics provided by Plastics Europe [31]. All the plastics constituting this database are referenced except the poly(vinyl choride) which is only used nowadays in building and construction for long-term applications. Based on this database we were able to identify more than 95% of plastic samples collected since only a few percent of the samples remain unidentified (less than 2%), whereas the most abundant types of plastic debris are PE, PP, PS and PA.

In all water strips analyzed, the large majority (from 73 to 79%) of plastic items found and subjected to FT-IR were made of polyolefins (Fig 2 and S2 Fig), basically polyethylene (PE, 62 to 76%) and polypropylene (PP, 7 to 14%), which agrees with what was found for surface waters in other regions [32, 11, 8]. PE and PP are light polymers (density: 0.85 to 0.97 g mL^1^; [33, 34] widely used in the packaging, typically single-use disposable products with a relatively short life time, rapidly ending up in waste and litter stream. Additionally, we found an important proportion of polyamides (PA, from 10 to 16%), a plastic family missing or reported at small proportions in other similar studies [35, 11, 8]. The best-known PA polymer is the nylon, the synthetic fiber commonly used for fishing lines. This suggests an extensive input of plastic from the fishing activity in the Mediterranean. On the other hand, the particular density of the Mediterranean waters could also explain the relatively high proportion of PA found in the surface waters. The density of the surface waters of the world ocean typically ranges from 1.02 to 1.03 g mL^-1^ [36], while PA density ranges from 1.02 to 1.15 g mL^-1^ [33, 34]. Therefore, only the less dense PA polymers could float in seawater. The Mediterranean waters, however, are among the most saline and densest waters (usually > 1.026 g mL^-1^), which broaden the range of PA polymers able to float. Another possible reason for the abundance of PA could be the entanglement of the nylon fibers with natural debris (e.g. pieces of wood, branches of Posidonia, bird feathers), generally abundant in our samples. The plastic composition in the coastal strip (< 1 km) diverged from other regions in the diversity of polymers found. The percentage of polystyrene (PS) and polyacrylic fibers (PAA) drastically decreased beyond 1-km from land. PAA fibers mainly have a textile origin, and largely enter the coastal waters via the drainage of our washing machines [35]. PAA density is generally higher than seawater (1.09–1.20 g mL^-1^ [34], but the shape and small size of these fibers favor the entanglement in other floating debris or their resuspension as result of the turbulent mixing [32].

**Fig 4:**
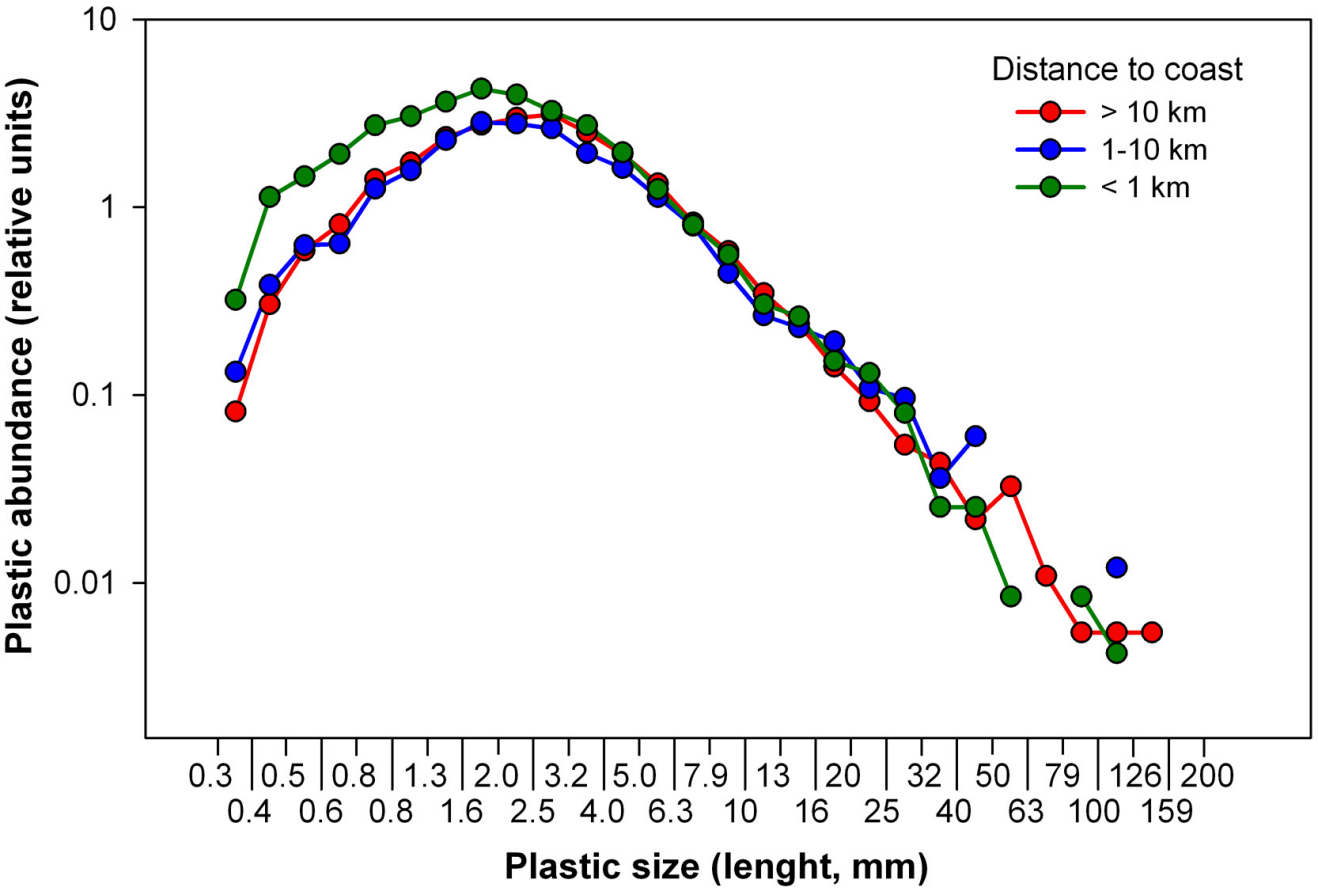
Size distribution of floating plastic debris in the Mediterranean Sea at different distances from land. Abundances are expressed as abundance relative to the total number of large items (> 10 mm) collected within each zone. The abundances were not normalized by width of the size-class interval in order to stress the differences between distributions along the size classes with the highest plastic abundances (note that the axis of relative abundance uses logarithmic scale). The size distributions normalized by both total number of large items and width of the size-class intervals are shown in S2 Fig. A total of 14,452 plastic items were used for this analysis, 3,812 of which were measured by Cózar et al. [15].

The analysis of the size distributions of plastic particles at different distances from land showed an increase in plastic abundance from large to small items and an important gap for the plastic of few millimeters and smaller (Fig 4 and S3 Fig). This is consistent with the general size distribution found by Cózar et al. [8] for ocean surface waters. However, we found a considerably higher relative abundance of small plastic particles (< 2 mm) in the 1-km water strip adjacent to coast. The plastic size distributions previously reported for offshore waters did not show such abundance of small particles [8, 21]. We hypothesize that this singularity in the nearshore plastic size distribution results from the combination of (i) an efficient removal of small fragments from the surface and (ii) a faster fragmentation of the plastic objects along the coastline, providing a faster flux of plastic towards small-size classes within short distances from coast. The existence of important sinks removing fragments of few millimeters (around 2 mm) was also inferred from the modeling of the plastic size distribution in the open ocean surface [8]. Several processes were suggested as responsible for this size-selective plastic loss, among them, biofouling was pointed out as particularly relevant and, although it was not systematically quantified in this study, the presence of biofouled particles was commonly observed in the samples. Surface to volume ratio increases exponentially as the plastics becomes smaller, until the ballasting effect by epiphytic growth exceeds the buoyancy of the smallest plastic particles [8, 12]. From the beaching of the nearshore plastic debris, they are exposed to solar radiation on land, reaching warmer temperatures and becoming brittle at considerably faster rates than plastic in water [37]. Moreover, the mechanical fragmentation derived from the wave breaking on the shores must accelerate the generation of small plastic fragments. Therefore in our study, changes in the shape of the size spectra from nearshore to offshore waters could be explained by an important removal of small plastics in conjunction with a fast fragmentation of the plastic debris along the coastline. However, we cannot rule out the possibility that these small fragments could also be more vulnerable to vertical transport [17, 18]. The area of 1-km water-strip of the Mediterranean Sea accounts for 1.8% of the total surface of Mediterranean Sea. Considering that the basin is extensively polluted by plastic, the contribution of the coastal floating plastic to the basin-scale load seem quantitatively irrelevant in relation to the available results. However the presence of high abundance of plastic items in the nearshore water strip, particularly of tiny (>2 mm) plastic items could generate other threats. The consequences could be relevant at ecological level as the build up of plastic along the shoreline is leading to particularly high plastic to plankton abundance ratios. This ratio averaged 0.03 ±1.40 for the two strips, < 1 km and 1–10 km from land, being one order of magnitude higher than those found in the NW of Sardinia Sea [38], 0.006 ±0.006 for sites beyond 10 km from coast (Fig 3B). Although ratios in our study were highly variable in the coastal strip (< 1 km) and not significantly different from offshore ratios (Mann-Whitney U, p = 0.488, N 30), they clearly reached the highest values (range: 0.00–0.11) in this water strip while were below 0.01 for sites >10 km from land. Coastal planktivorous animals with low prey selectivity show a high probability to ingest plastic particles. Commercial bivalves are a meaningful example. Significant quantities of microplastics are commonly reported worldwide in coastal waters in these filter feeders [39, 40]. Moreover, the aged microplastic concentrates pollutants that could be absorbed by marine life and biomagnified along the marine food web from the planktivorous fauna [41, 42]. Therefore, the conjunction of high concentrations of microplastics, as reported here, and pollutants released from industrial, agricultural and urban land-based sources [43] could often take place in the nearshore waters and create an environment conducive to the plastic-mediated transfer of pollutants. The coastal plastic accumulation in the Mediterranean has also a perceptible impact on the touristic attractiveness of the shoreline. The removal of floating patches of debris by collection boats is a necessary work in many Mediterranean beaches for a long time now.

## Conclusions and Final Remarks

In the present work, we show a coherent large-scale pattern, spanning from hundreds of meters to hundreds of kilometers for the distribution of floating plastic debris in the Mediterranean Sea in spite of the high spatial heterogeneity at small scales (around tens of kilometers and less). The 1-km water strip adjacent to land was shown to be a zone with elevated plastic abundance, high diversity of polymers and higher proportion of small-sized fragments (< 2.5 mm). Our data are focused on the Northwestern Mediterranean coast, where high concentrations of plastic debris were previously observed [44] and the density of population and recreational activities is relatively high. The extent and relevance of the coastal plastic accumulation varies with the regional hydrodynamics and the nearby population density. In our study area the continental shelf is between 1 and 5 km wide, and the permanent Northern Current flows alongside the coastline, few kilometers away from land [45]. On one hand, the energetic Northern Current and its frontal system, characterized by a strong density gradient, could help to concentrate plastic nearshore, on the other hand, the current itself may contribute to the dispersal of floating debris (S4 Fig). Yet it is likely that a higher concentration of plastic found closer to the coast and an increase in offshore areas is a common feature of marine plastic distribution.

Boundary currents sweeping the shelf border of the basin, as the Northern Current, are common in the Mediterranean [46, 22] as well as in other ocean basins [3]. At long distances from land (tens or hundreds kilometers), far from the influence of perimetric currents, the results showed a high occurrence of samples with abundant plastic. This pattern is also observed at large scale in the oceanic basins, where the offshore areas of low dispersion and surface convergence are identified as accumulation regions for the floating plastic [6, 8, 9]. In the Mediterranean Sea, the potential structures of plastic accumulation such as the centers of anticyclonic gyres and isolated eddies, are often instable structures, hampering the formation of stable plastic accumulations zones [22]. The periodic strong wind stress, that mixes and spreads seawards riverine waters with land-based plastics, associated with the complex circulation patterns may explain the lack of permanent open sea aggregation zones. Yet, our results suggest that the hydrodynamic features of the central part of the basin could retain temporarily high plastic concentrations and set up a basin-scale rising trend in plastic pollution toward offshore regions. The additional input of plastic trash from the heavy offshore shipping activities could also contribute to the plastic accumulations in high seas, although this input of fresh debris had no apparent effect on the plastic size distribution offshore (Fig 4). Surface coastal waters remains widely unexplored, and therefore are unaccounted for the global estimates of floating plastic load [8, 9]. The modeling [4] of the floating debris transport released from the world coastlines showed that from 36% to 40% of debris sat along the coastline after 30 year of simulation, suggesting that the coastal plastic accumulations could become relevant at global scale.

Coastal areas are subjected to fresh inputs of anthropogenic litter from land and riverine watercourses, mainly carried by winds, urban waterways or directly discharged into coastal waters [47]. Our results showed a relationship between plastic concentration nearshore and coastal populations (Fig 3A). Despite a large heterogeneity, the relationship we found opens a way to model the variability and fate of coastal plastic although more data are still required. Other coastal reservoirs of plastic as beaches and coastal seafloor should be also assessed taking into account specific samplings and assessments on floating micro and macro-plastic fraction.

The concern about the marine plastic pollution is generating multiple initiatives to prevent, mitigate and monitor this emerging problem. In Europe, the Marine Strategy Framework Directive (MSFD) recognizes marine litter as one of the descriptors for the environmental state of the European seas [48]. However, our limited understanding concerning input, distribution and transformation of the marine plastic debris make this descriptor difficult to deploy. Accurate assessment of oceanic plastic debris including distribution and transport of plastics is among research priorities to mitigate impacts of this pollution on marine wildlife and threatened species [49]. The present work points at the coastal waters and the tiny plastics as hot spots for the managing and monitoring actions given the potential ecological, sanitary and economic impacts of this kind of pollution.

## Supporting Information

S1 Fig Comparison of floating plastic debris size distributions > 10 km offshore in this study (n = 712) and in Cózar et al. (n = 3756). The size distributions were in a good match, with a smoother shape for the sampling with higher data number.
(TIFF)

S2 Fig FTIR spectra of the most frequent plastic debris -(a): Polyethylene PE; (b): polypropylene PP; (c): polyamides PA and (d): polystyrene PS. (TIFF)

S3 Fig Size distribution of floating plastic debris in the Mediterranean Sea at different distances from land expressed in abundances normalized by the width of the size classes. In contrast to Fig 4, here the abundances are divided by the total number of large items (> 10 mm) collected within each zone as well as the width of the size-class intervals (in mm). The plastic count in each size class is independent of the bin width used, allowing for the comparison of plastic densities along the size spectrum [8].
(TIFF)

S4 Fig Direction and intensity of surface currents in the NW Mediterranean sea. Maps from May and August 2013. Data derived from the model MARS 3D. Units in are cm/s. with arrows color-coded with speeds. Reprinted from [http://www.previmer.org] under a CC BY license, with permission from [PREVIMER].
(TIF)
