# Supplementary figures and images for: Changes in the Floating Plastic Pollution of the Mediterranean Sea in Relation to the Distance to Land
Source: PLoS One. 2016 Aug 24;11(8):e0161581. doi: 10.1371/journal.pone.0161581 (PMC4996504; doi:10.1371/journal.pone.0161581)

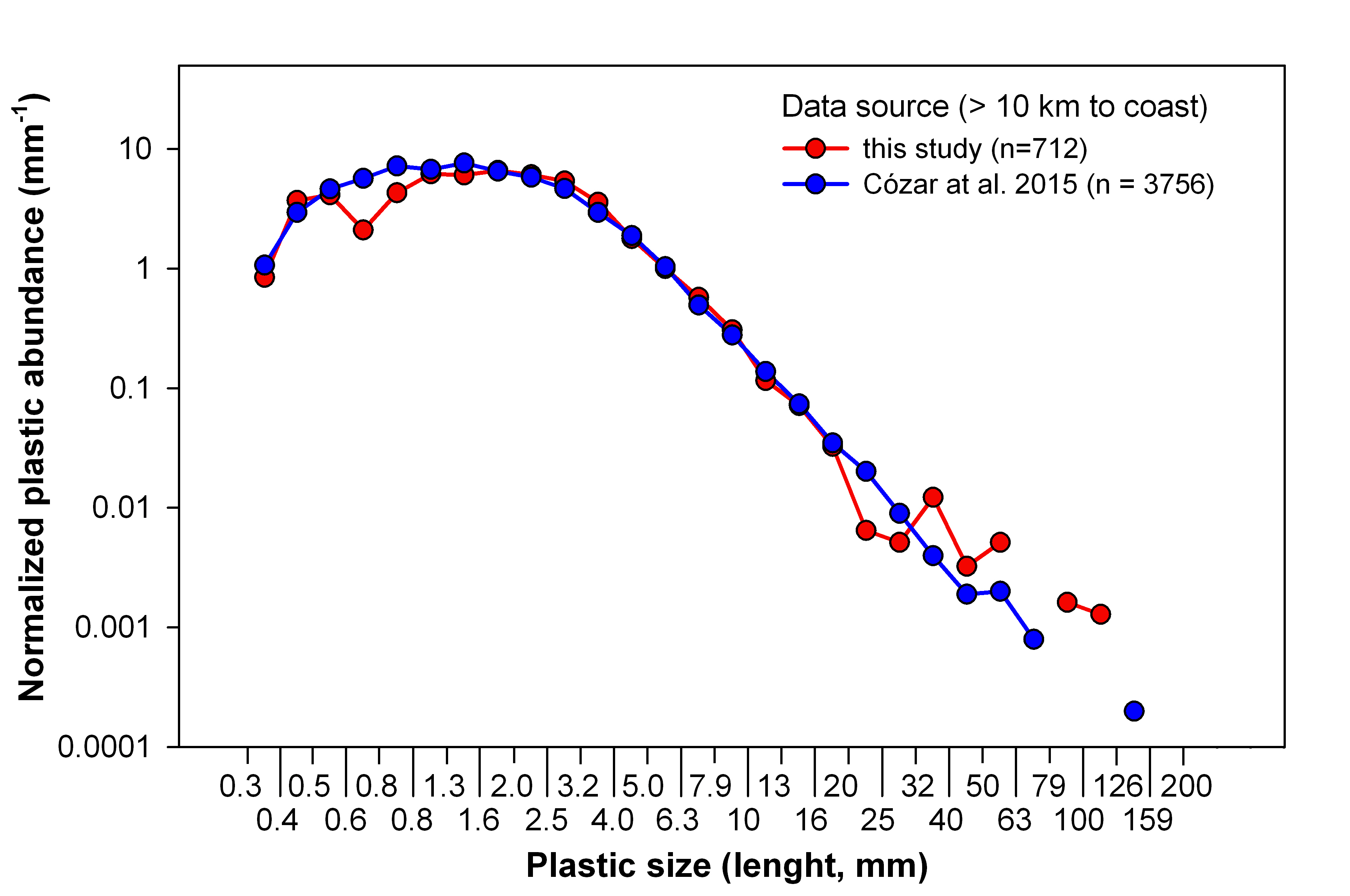

Supplement: S1 Fig — The size distributions were in a good match, with a smoother shape for the sampling with higher data number. (TIFF) [file pone.0161581.s001.tiff]

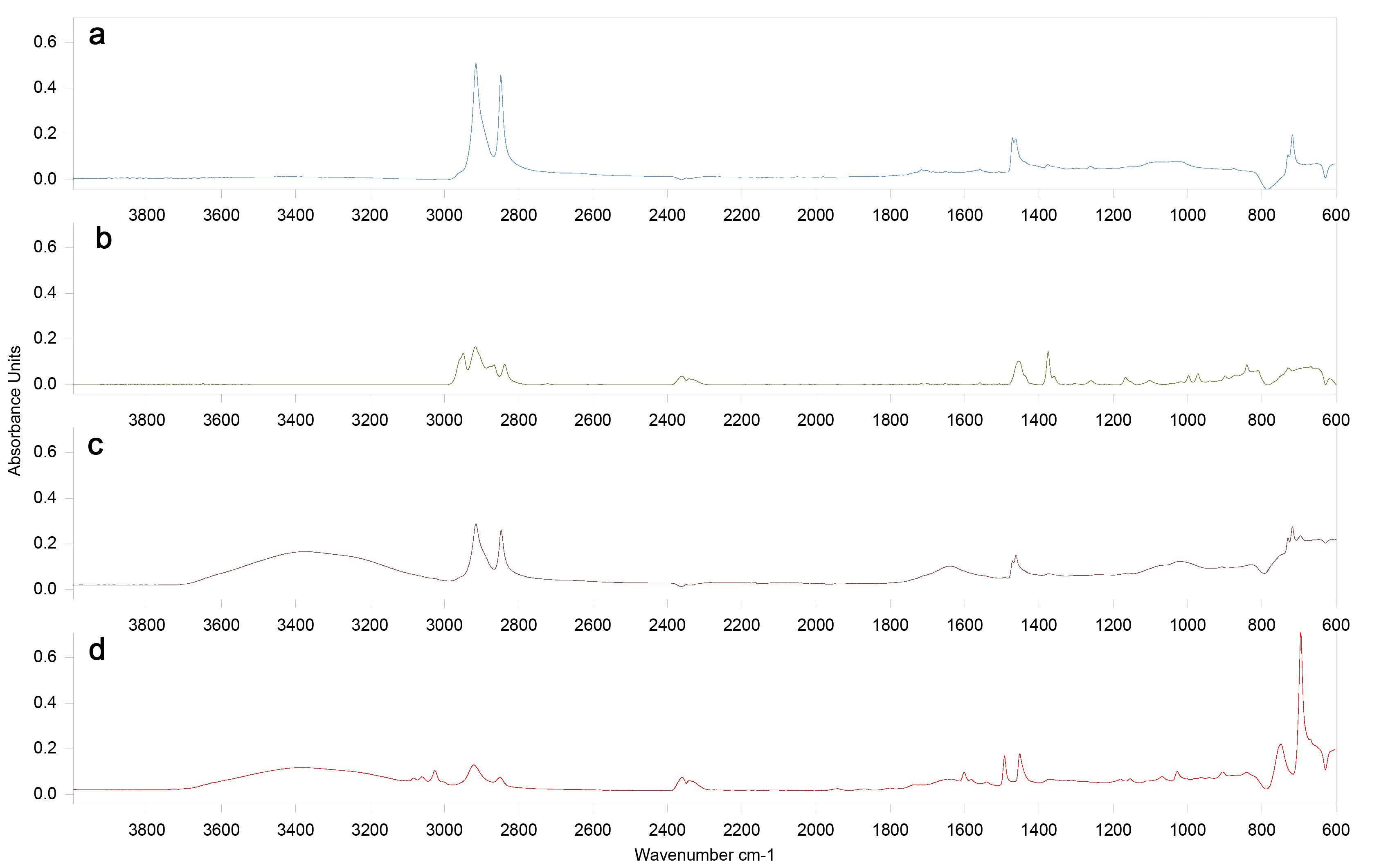

Supplement: S2 Fig — (TIFF) [file pone.0161581.s002.tiff]

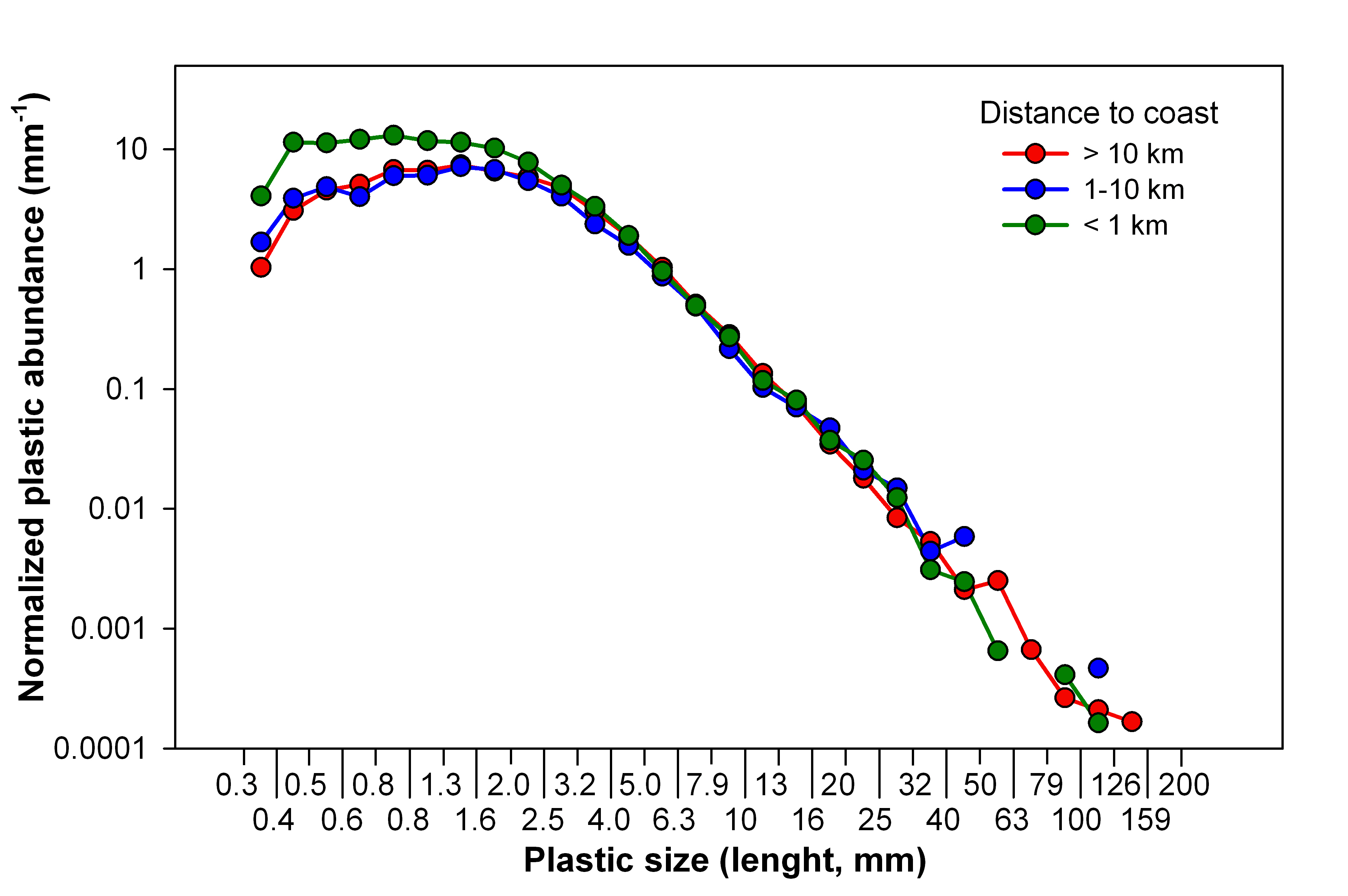

Supplement: S3 Fig — In contrast to Fig 4, here the abundances are divided by the total number of large items (> 10 mm) collected within each zone as well as the width of the size-class intervals (in mm). The plastic count in each size class is independent of the bin width used, allowing for the comparison of plastic densities along the size spectrum [8]. (TIFF) [file pone.0161581.s003.tiff]

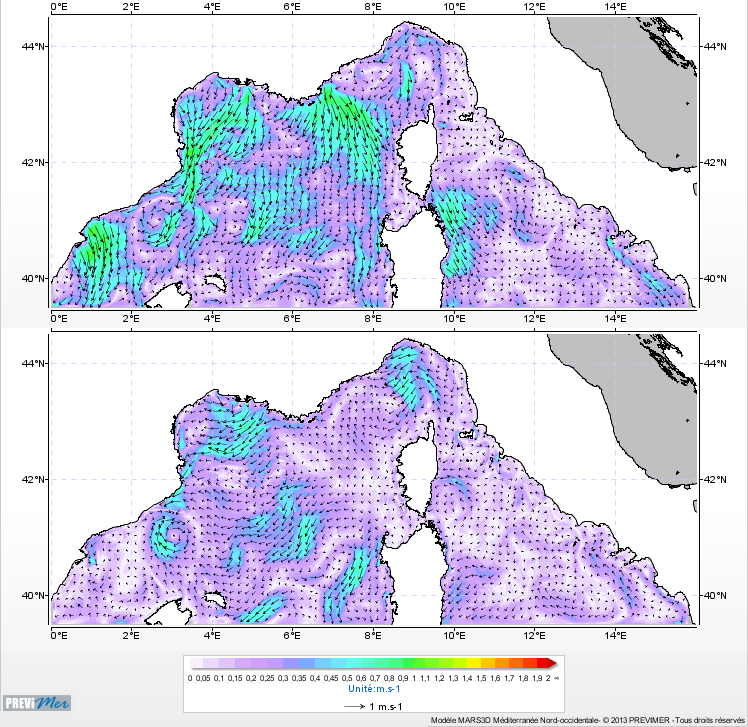

Supplement: S4 Fig — Maps from May and August 2013. Data derived from the model MARS 3D. Units in are cm/s. with arrows color-coded with speeds. Reprinted from [http://www.previmer.org] under a CC BY license, with permission from [PREVIMER]. (TIF) [file pone.0161581.s004.tif]
